# MORE: a multi-omics data-driven hypergraph integration network for biomedical data classification and biomarker identification

**DOI:** 10.1093/bib/bbae658

**Published:** 2024-12-18

**Authors:** Yuhan Wang, Zhikang Wang, Xuan Yu, Xiaoyu Wang, Jiangning Song, Dong-Jun Yu, Fang Ge

**Affiliations:** School of Computer Science and Engineering, Nanjing University of Science and Technology, 200 Xiaolingwei, Nanjing 210094, China; Biomedicine Discovery Institute and Department of Biochemistry and Molecular Biology, Monash University, Wellington Rd, Clayton, Melbourne, VIC 3800, Australia; Department of Computer Science, City University of Hong Kong, Kowloon, Hong Kong 999077, China; Biomedicine Discovery Institute and Department of Biochemistry and Molecular Biology, Monash University, Wellington Rd, Clayton, Melbourne, VIC 3800, Australia; Biomedicine Discovery Institute and Department of Biochemistry and Molecular Biology, Monash University, Wellington Rd, Clayton, Melbourne, VIC 3800, Australia; Monash Data Futures Institute, Monash University, Wellington Rd, Clayton, Melbourne, VIC 3800, Australia; School of Computer Science and Engineering, Nanjing University of Science and Technology, 200 Xiaolingwei, Nanjing 210094, China; State Key Laboratory of Organic Electronics and Information Displays & Institute of Advanced Materials (IAM), Nanjing University of Posts & Telecommunications, 9 Wenyuan, Nanjing 210023, China

**Keywords:** comprehensive hyperedge group, multi-omics hypergraph encoding module, multi-omics self-attention mechanism, identify disease-related biomarkers

## Abstract

High-throughput sequencing methods have brought about a huge change in omics-based biomedical study. Integrating various omics data is possibly useful for identifying some correlations across data modalities, thus improving our understanding of the underlying biological mechanisms and complexity. Nevertheless, most existing graph-based feature extraction methods overlook the complementary information and correlations across modalities. Moreover, these methods tend to treat the features of each omics modality equally, which contradicts current biological principles. To solve these challenges, we introduce a novel approach for integrating multi-omics data termed Multi-Omics hypeRgraph integration nEtwork (MORE). MORE initially constructs a comprehensive hyperedge group by extensively investigating the informative correlations within and across modalities. Subsequently, the multi-omics hypergraph encoding module is employed to learn the enriched omics-specific information. Afterward, the multi-omics self-attention mechanism is then utilized to adaptatively aggregate valuable correlations across modalities for representation learning and making the final prediction. We assess MORE’s performance on datasets characterized by message RNA (mRNA) expression, Deoxyribonucleic Acid (DNA) methylation, and microRNA (miRNA) expression for Alzheimer’s disease, invasive breast carcinoma, and glioblastoma. The results from three classification tasks highlight the competitive advantage of MORE in contrast with current state-of-the-art (SOTA) methods. Moreover, the results also show that MORE has the capability to identify a greater variety of disease-related biomarkers compared to existing methods, highlighting its advantages in biomedical data mining and interpretation. Overall, MORE can be investigated as a valuable tool for facilitating multi-omics analysis and novel biomarker discovery. Our code and data can be publicly accessed at https://github.com/Wangyuhanxx/MORE.

## Introduction

Significant advancements in diverse high-throughput sequencing methods, e.g. DNA nanosphere sequencing, mRNA expression (mRNA), DNA methylation (meth), and miRNA expression (miRNA), have fundamentally revolutionized various biological analyses and facilitated biological discoveries [1, 2]. Current research has demonstrated diverse omics data contains both shared and unique knowledge of various biological processes [3, 4]. Combining multi-omics data enables deeper insights of analyses, leading to better clinical decisions and improved disease treatment [5, 6]. Several studies also highlight that multi-omics integration enhances the accuracy of disease prediction when compared to single-omics approaches [7–9]. Although a variety of approaches have been proposed, most of them only consider the commonalities, ignoring the complementarities across omics modalities. Consequently, there is a pressing need for an effective integration method to analyze and explore interactive and complementary information in multiple omics data.

Existing multi-omics integration approaches are categorized into unsupervised and supervised learning ones. Unsupervised learning approaches encode integrated multiple omics data into low-dimensional feature embeddings to perform classification and clustering tasks [10, 11]. However, due to the lack of labeled information for supervised training, the experiment results are usually unsatisfactory. In recent years, numerous studies have focused on employing supervised learning to explore various biological processes and mechanisms. For instance, Van De Wiel *et al*. [7] proposed an adaptive group-regularized ridge regression technique that incorporates methylation microarray data alongside curated annotations for the classification of cervical cancer. Moreover, Singh *et al*. [3] proposed Data Integration Analysis for Biomarker discovery using Latent cOmponents (DIABLO), which extends sparse generalized canonical correlation analyses to supervised learning. DIABLO is capable of both discriminating different phenotypic groups and identifying the common information across different omics modalities. However, the simple linear correlation methods do not apply to complex disease studies.

Over the past few years, deep learning techniques have been widely utilized in integrating multiple omics data because of its powerful capability to learn nonlinear relationships across modalities [12, 13]. For example, Wang *et al*. [14] proposed Multi-Omics Graph cOnvolutional NETworks (MOGONET), which uses independent graph convolutional networks (GCNs) [15] to encode various omics datasets. Notably, it constructs a multiple omics intersection tensor to explore cross-omics correlations for effectively integrating multiple omics data. The limitation lies that an employed GCN can only model pairwise correlations between nodes, which is not optimal for biological analysis with complex correlations. Dong *et al*. [16] proposed Multi-Omics Graph Learning and Attention Mechanism (MOGLAM), which utilizes dynamic GCNs (FSDGCNs) to select the feature and omic-integrated representation learning (OIRL) to integrate multi-omics data. MOGLAM also builds an advanced sample similarity network, leading to the extraction of richer and more informative embeddings. Gong *et al*. [17] introduced Multi-Omics Attention Deep Learning Network (MOADLN), an approach that integrates multi-omics data using the self-attention mechanism [18] and a multi-omics association discovery network. However, MOGONET, MOGLAM, and MOADLN focus solely on the influence between the samples within each individual omics modality when constructing the sample similarity network. This strategy neglects the complementary information across different omics modalities, which can potentially result in inferior network performance.

Recently, hypergraphs have become an effective tool for modeling and exploring complicated correlations across different data modalities in numerous applications [19, 20]. Distinct from previous graph representations [15, 21], hypergraphs can utilize degree-free hyperedges to encode higher-order data correlations, which is a more efficient way to model complex correlations of nodes and enable more complex data analysis. To solve the aforementioned problems in multi-omics analysis, we introduce an innovative multi-omics integration approach, MORE. Specifically, MORE is a model comprising the Multi-Omics Hypergraph Encoding (MOHE) module to learn more representative omics-specific features and the Multi-Omics Self-Attention (MOSA) module to integrate valuable information across modalities to make the final prediction. Firstly, we constructed a hyperedge group for each omics modality by extensively mining the potential correlations within each omics modality. Subsequently, we fused the hyperedge groups from different modalities to construct a comprehensive hyperedge group. Next, the hypergraph, along with the features from each omics modality, was inputted into the MOHE module for omics-specific knowledge learning. Therefore, the MOHE module considers the correlations within and across different omics modalities, which could ensure a more discriminative omics-specific representation with richer information. Considering the varied contributions of different omics modalities to the final classification, the MOSA module was employed to adaptively integrate these features based on generated attention coefficients.

In the Introduction section, we proposed an innovative multi-omics integration method termed MORE. Then, in the Materials section, we introduced the datasets used and the data preprocessing method. In the Methods section, we described the construction of the different modules of the model in detail. In the Results and Discussions section, we demonstrated the effectiveness and potential applications of MORE based on extensive experiments. Benchmarking experiments demonstrated that MORE outperformed other multi-omics integration methods. Moreover, ablation experiments confirmed the essential contribution of the MOHE and MOSA modules to the performance of MORE. In the Identifying Biomarkers Using MORE section, we showed that MORE could identify important biomarkers relevant to biomedical problems, indicating its data mining and interpretation capabilities. Finally, we summarized the advantages and limitations of the model in the Conclusion section. Overall, MORE attains better performance than other existing advanced multiple omics integrated methods and can be potentially applied as a useful tool to facilitate community-wide efforts in multi-omics data analysis.

## Materials

### Datasets

Three datasets were used in this study: The Religious Orders Study and the Rush Memory and Aging Project (ROSMAP) for Alzheimer’s disease (AD), normal control (NC) classification, invasive breast carcinoma (BRCA) for invasive breast carcinoma PAM50 subtype classification, and glioblastoma (GBM) for glioblastoma subtype classification. All datasets comprise omics data on mRNA, meth, and miRNA.

ROSMAP was retrieved from AMP-AD Knowledge Portal [14, 22], while BRCA was extracted from The Cancer Genome Atlas Program (TCGA) via Broad GDAC Firehose [14]. PAM50 identifies five molecular subtypes of breast cancer, including normal-like, basal-like, HER2-enriched, Luminal A, and Luminal B [23]. BRCA subtype data from PAM50 were accessed through TCGAbiolinks [24]. The GBM dataset was obtained from the benchmark cancer datasets [25]. The GBM dataset has four different subtypes including the proneural, classical, mesenchymal, and neural [26, 27]. A detailed description of the three datasets regarding disease categories and the sample amounts is provided in Table 1.

**Table 1:** Detailed information of the datasets in terms of disease categories and the number of samples.

Dataset	Categories	Number of features for training mRNA, meth, and miRNA
ROSMAP	NC: 169, AD: 182	200, 200, 200
BRCA	Normal-like: 105, Basal-like: 128, HER2-enriched: 44, Luminal A: 385, Luminal B: 146	1000, 1000, 503
GBM	Proneural: 66, Classical: 55, Mesenchymal: 66, Neural: 39	1000, 1000, 534

### Preprocessing

Appropriate preprocessing of omics data is crucial to eliminate experimental errors and noise. First, we removed features with missing values (identified as NaN). Afterward, considering that probes for DNA methylation data might correspond to multiple genes, the probes corresponding to a single gene were reserved to ensure the data sensitivity. Subsequently, features with low variances or no signal were also filtered out. Particularly, different variance filtering thresholds were used for multiple omics data. For mRNA and meth data, the variance thresholds were set to 0.1 and 0.001, respectively. The miRNAs’ amount in the expression data is much fewer than that in the other two modalities; thereby, only features with zero variance were filtered out.

Although numerous preprocessing operations were employed, the high-dimensional omics data might still include needless information that could negatively impact the model performance. Consequently, the analysis of variance (ANOVA) was employed to further refine feature selection. ANOVA *F*-values were calculated for every omics data to determine the variance between categories. Subsequently, we selected the features that significantly varied between different categories. Eventually, we standardized all of omics data to [0,1].

## Methods

**Figure 1:**
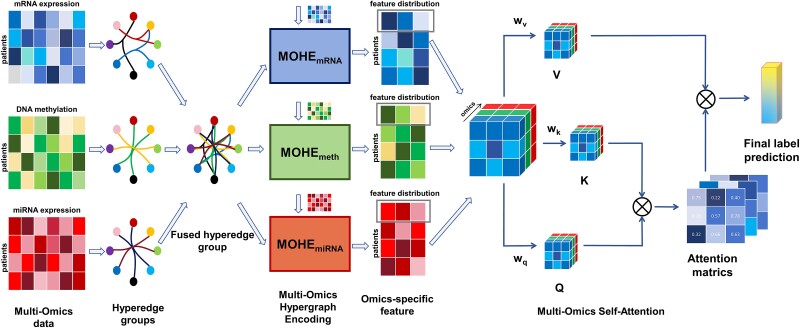
Overview of the proposed MORE for multi-omics analysis. The framework comprises the fused hyperedge group generation process, the multi-omics hypergraph encoding process, and the multi-omics self-attention process. The final prediction is based on the aggregated multi-omics features. For the binary classification task, the final prediction of the model represents AD and NC. For the multi-class classification tasks, the final prediction of the model represents different subtypes of the disease.

This section introduces the proposed MORE method in detail. As illustrated in Fig. 1, MORE builds upon two main modules: MOHE and MOSA. The MOHE module is used to extract omics-specific knowledge and simultaneously reveal the correlations within and across omics modalities in the latent feature space. The MOSA module further efficiently integrates multi-omics features to generate the final prediction.

### Multi-omics hypergraph construction and representation learning

The hypergraph neural network stands out in comparison with previous graph representations, which can effectively utilize degree-free hyperedges to encode complex correlations across data modalities [20, 28]. Generally, the hypergraph is represented as $\mathrm{G}=\left(\mathrm{V},\mathrm{E},\mathrm{W}\right)$, $\mathrm{V}$ and $\mathrm{E}$ denote the vertex and hyperedge set. The diagonal matrix of edge weights $\mathrm{W}$ assigns a weight to each hyperedge. $\mathrm{G}$ can be represented with a $\mid \mathrm{V}\mid \times \mid \mathrm{E}\mid$ incidence matrix $\mathrm{H}$, as follows:


(1) \begin{equation*} h\left(v,e\right)=\left\{\begin{array}{@{}l}1,\kern0.75em \mathrm{if}\kern0.5em v\in e\\{}0,\kern0.75em \mathrm{if}\kern0.5em v\notin e\end{array}\right. \end{equation*}

where $v\in e$ denotes that node $v$ is in the hyperedge $e$. For $v\in \mathrm{V}$, $d(v)={\sum}_{e\in \mathrm{E}}w(e)h\left(v,e\right)$ represents its degree. For $e\in \mathrm{E}$, $d(e)={\sum}_{v\in \mathrm{V}}h\left(v,e\right)$ represents its degree. Additionally, ${\mathrm{D}}_v$ and ${\mathrm{D}}_e$ represent the diagonal matrices of the vertex degrees and the edge degrees, respectively.

**Figure 2:**
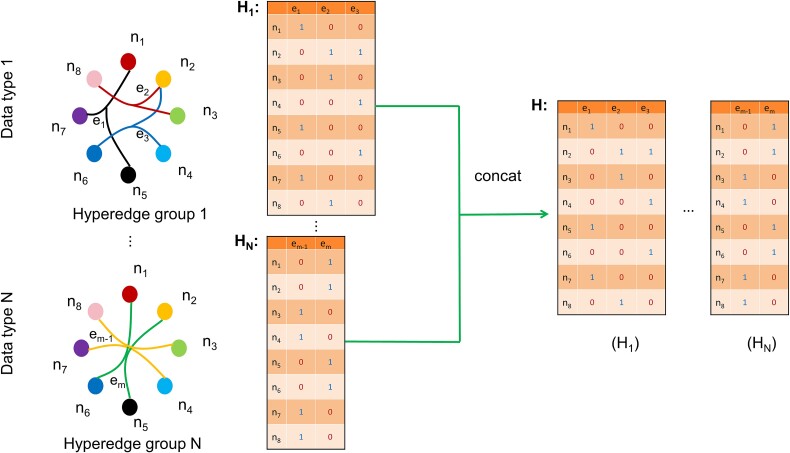
Fusion of hyperedge groups by combining the incidence matrices across modalities.

The following subsection introduces the generation process of ${\boldsymbol{H}}_{\mathrm{mRNA}}$, ${\boldsymbol{H}}_{\mathrm{meth}}$, and ${\boldsymbol{H}}_{\mathrm{miRNA}}$ in detail. For each vertex, we computed its distance from all other vertices and constructed its hyperedge by connecting the $k$ nearest ones. Here, $k$ is an important hyper-parameter that represents the average amount of vertices connected by each hyperedge (also itself). If the two vertices are connected, the value of the corresponding position in the incidence matrix will be defined as 1, otherwise 0. We obtained ${\boldsymbol{H}}_{\mathrm{mRNA}}$, ${\boldsymbol{H}}_{meth}$, and ${\boldsymbol{H}}_{\mathrm{miRNA}}$ for the three omics modalities in this method. As shown in Fig. 2, a hypergraph can jointly employ multi-omics correlations for hyperedge group fusion by combining the incidence matrices [20]. To create the final multi-omics hypergraph $\mathrm{G}=\left(\mathrm{V},\mathrm{E},\mathrm{W}\right)$, we concatenated the three incidence matrices from the three omics modalities. The global incidence matrix $\mathrm{H}\in{R}^{N\times 3N}$ can be represented as follows:


(2) \begin{equation*} \mathrm{H}=\mathrm{Concat}\left[{\boldsymbol{H}}_{\mathrm{mRNA}},{\boldsymbol{H}}_{\mathrm{meth}},{\boldsymbol{H}}_{\mathrm{miRNA}}\right] \end{equation*}

Furthermore, $\mathrm{G}\in{R}^{N\times N}$ can be defined as follows:


(3) \begin{equation*} \mathrm{G}={\mathrm{D}}_v^{-1/2}{\mathrm{H}\mathrm{WD}}_e^{-1}{\mathrm{H}}^{\top }{\mathrm{D}}_v^{-1/2} \end{equation*}

Eventually, we can construct a hypergraph convolutional layer$f\left(\mathrm{X},\mathrm{W},\Theta \right)$:


(4) \begin{equation*} {\mathrm{X}}^{\left(l+1\right)}=\sigma \left({\mathrm{D}}_v^{-1/2}{\mathrm{H}\mathrm{WD}}_e^{-1}{\mathrm{H}}^{\top }{\mathrm{D}}_v^{-1/2}{\mathrm{X}}^{(l)}{\Theta}^{(l)}\right) \end{equation*}

where ${\mathrm{X}}^{(l)}\in{R}^{N\times C}$ represents the input feature of a hypergraph at an $l$ layer, ${\mathrm{X}}^{(0)}=\mathrm{X}$, $\Theta$ optimized throughout the training phase, $\sigma$ is the nonlinear activation function.

### Multi-omics integration learning

Existing graph-based multi-omics integration methods generally treat features from different modalities equally. Nevertheless, various omics modalities may offer unique contributions to different biological analysis processes [16]. Given the significant capability of the self-attention mechanism in representation learning [29–32], we employed it to adaptatively establish the correlations across omics modalities.

For the patient $j$, its feature matrix is represented as ${\hat{\mathrm X}}_j=\left[{\hat{x}}_j^{(1)},{\hat{x}}_j^{(2)},...,{\hat{x}}_j^{(I)}\right]$, where ${\hat{x}}_j^{(i)}$ denotes the feature vector of the *i*-th omics. Subsequently, the Query, Key, and Value can be generated by ${\mathrm{Q}}_j={w}_q{\hat{\mathrm X}}_j+{b}_q=\left[{q}_j^{(1)},{q}_j^{(2)},...,{q}_j^{(I)}\right]$, ${\mathrm{K}}_j={w}_k{\hat{\mathrm X}}_j+{b}_k=\left[{k}_j^{(1)},{k}_j^{(2)},...,{k}_j^{(I)}\right]$, and ${\mathrm{V}}_j={w}_v{\hat{\mathrm X}}_j+{b}_v=\left[{v}_j^{(1)},{v}_j^{(2)},...,{v}_j^{(I)}\right]$, respectively; $w$ and $b$ correspond to the optimizable parameters. The attention matrix among multi-omics can be defined as:


(5) \begin{equation*} {\mathrm{A}}_j\left(m,n\right)=\frac{\exp \left[{q}_j^{(m)}\cdot{\left({k}_j^{(n)}\right)}^{\top }/\sqrt{d_f}\right]}{\sum_{n=1}^I\exp \left[{q}_j^{(m)}\cdot{\left({k}_j^{(n)}\right)}^{\top }/\sqrt{d_f}\right]} \end{equation*}

where ${\mathrm{A}}_j\left(m,n\right)$ indirectly reflect the correlations between *m*-th and *n*-th omics in sample$j$. With the attention matrix ${\mathrm{A}}_j$, the final aggregation process can be formulated as follows:


(6) \begin{equation*} {\hat{\mathrm V}}_j={\mathrm{A}}_j\cdot{\mathrm{V}}_j^{\top } \end{equation*}

The multihead operation was also utilized in this feature learning process, which aims to achieve an enriched feature collection from multiple perspectives. The final representation can be formulated as follows:


(7) \begin{equation*} {\hat{\mathrm V}}_j^{cat}= Concat\left[{\hat{\mathrm V}}_j^{(1)},{\hat{\mathrm V}}_j^{(2)},...,{\hat{\mathrm V}}_j^{(Z)}\right] \end{equation*}

where $Z$ refers to the number of heads. Eventually, a linear classification layer was employed to classify the patients using the generated representations.

### Model optimization

To enhance omics-specific information and improve the overall network performance, we introduced a two-step learning strategy with the first step only optimizing the omics-specific MOHEs and the second step optimizing the whole network. The cross-entropy loss function was adopted for optimization. In the first training step, the loss function tailored to the *i*-th omics modality can be formulated as follows:


(8) \begin{equation*} {L}_{\mathrm{omics}-\mathrm{specific}}^{(i)}=\sum_{j=1}^N{L}_{\mathrm{CE}}\left({p}_j^i,{y}_j\right) \end{equation*}

where ${y}_j$ represents true label, ${p}_j^i$ is the prediction from the omics-specific layer, while $N$ refers to the sample amounts, respectively. During the second phase, the total loss function is shown as follows:


(9) \begin{equation*} L=\sum_{i=1}^3{L}_{\mathrm{omics}-\mathrm{specific}}^{(i)}+{L}_{\mathrm{multi}-\mathrm{omics}} \end{equation*}

To demonstrate the superiority of the training strategy, we also performed experiments using an end-to-end training strategy. Experimental results and details are shown in the section Model Performance Based on Different Training Strategies.

## Results and discussions

### Evaluation metrics

In this study, each dataset was partitioned into nonoverlapping two subsets: 70% training and 30% testing. For the binary classification task, the performance was assessed using multiple performance metrics including accuracy (ACC), F1 score (F1), and the area under the receiver operating characteristic curve (AUC). For the multi-class classification tasks, we utilized ACC, the weighted F1 (F1_weighted), and the macro-averaged F1 (F1_macro). There is a thorough description of the performance assessment metrics in Text S1. The implementation details of MORE are given in Text S2. The experiment was conducted five times on each dataset, with the average of these five trials reported as the final performance.

### Comparison with other multi-omics integration methods

We evaluated MORE with eight superior multi-omics integration approaches: (i) K-Nearest Neighbors (KNN) [33, 34], (ii) Support Vector Machine (SVM) [35, 36], (iii) Random Forest (RF) [37, 38], (iv) eXtreme Gradient Boosting (XGBoost) [39, 40], (v) fully connected Neural Network (NN) [41, 42], (vi) MOGONET [14], (vii) MOGLAM [16], and (viii) MOADLN [17]. The preprocessed multi-omic data were directly concatenated and utilized as input for KNN, SVM, RF, XGBboost, and NN.

From Table 2, we concluded that MORE attained the best performance on three classification tasks compared to other superior multiple omics integration approaches. For example, on ROSMAP, AUC of MORE was 1.7% higher than the second-best MOADLN. On BRCA, ACC of MORE was 1.3% higher than the second-best MOADLN. It is noteworthy that on the GBM dataset, even with small-size training samples, MORE still outperformed other machine learning and deep learning approaches, highlighting its generalization and robustness capability.

**Table 2:** Classification performance of all methods on three datasets.

Dataset		Evaluation metrics		
ROSMAP	Method	ACC	F1	AUC
	KNN	0.661 ± 0.038	0.682 ± 0.029	0.701 ± 0.037
	SVM	0.787 ± 0.015	0.791 ± 0.017	0.790 ± 0.016
	RF	0.735 ± 0.029	0.741 ± 0.030	0.815 ± 0.029
	XGBoost	0.775 ± 0.039	0.784 ± 0.040	0.846 ± 0.036
	NN	0.771 ± 0.012	0.779 ± 0.014	0.842 ± 0.017
	MOGONET	0.814 ± 0.022	0.819 ± 0.021	0.882 ± 0.017
	MOGLAM	0.816 ± 0.014	0.822 ± 0.013	0.885 ± 0.017
	MOADLN	0.816 ± 0.014	0.823 ± 0.019	0.886 ± 0.018
	MORE	0.829 ± 0.018	0.836 ± 0.017	0.903 ± 0.010
**BRCA**	**Method**	**ACC**	**F1_weighted**	**F1_macro**
	KNN	0.741 ± 0.013	0.710 ± 0.018	0.662 ± 0.023
	SVM	0.737 ± 0.017	0.696 ± 0.021	0.637 ± 0.028
	RF	0.755 ± 0.010	0.741 ± 0.011	0.661 ± 0.013
	XGBoost	0.779 ± 0.007	0.771 ± 0.007	0.714 ± 0.012
	NN	0.770 ± 0.020	0.754 ± 0.031	0.690 ± 0.032
	MOGONET	0.815 ± 0.017	0.812 ± 0.016	0.742 ± 0.023
	MOGLAM	0.819 ± 0.012	0.813 ± 0.014	0.750 ± 0.014
	MOADLN	0.822 ± 0.018	0.816 ± 0.018	0.755 ± 0.019
	MORE	0.835 ± 0.020	0.820 ± 0.023	0.768 ± 0.021
**GBM**	**Method**	**ACC**	**F1_weighted**	**F1_macro**
	KNN	0.665 ± 0.033	0.618 ± 0.031	0.555 ± 0.037
	SVM	0.702 ± 0.019	0.637 ± 0.018	0.580 ± 0.017
	RF	0.670 ± 0.023	0.643 ± 0.026	0.590 ± 0.030
	XGBoost	0.709 ± 0.026	0.692 ± 0.027	0.634 ± 0.029
	NN	0.707 ± 0.028	0.694 ± 0.023	0.667 ± 0.024
	MOGONET	0.740 ± 0.025	0.727 ± 0.021	0.702 ± 0.021
	MOGLAM	0.741 ± 0.024	0.733 ± 0.023	0.725 ± 0.023
	MOADLN	0.743 ± 0.022	0.734 ± 0.025	0.727 ± 0.019
	MORE	0.762 ± 0.027	0.755 ± 0.025	0.736 ± 0.024

### Ablation study

Herein, we evaluated and discussed the performance of proposed modules during the multi-omics analysis process. Extensive ablation experiments were performed to compare the performance of MORE with its three variants: (i) NN_NN: NNs for multiple omics feature learning and integration. (ii) NN_MOSA: NN for multiple omics feature learning and MOSA for integration. (iii) MOHE_NN: MOHE for multiple omics feature learning and NN for integration. In the ablation experiments, all NNs had the same number of layers as the replaced modules.

From Table 3 and Table S1, it was evident that MORE performed better than all three model variants in every task. For example, ACC of MORE was 0.9% higher than that of MOHE_NN, 3.5% higher than that of NN_MOSA, and 4.3% higher than that of NN_NN on the ROSMAP dataset. Although the ACC of MOHE_NN classification tasks was close to that of MORE on the ROSMAP dataset, MORE consistently yielded better metrics than MOHE_NN across all metrics for all tasks. Notably, MOHE_NN performed better than NN_MOSA and NN_NN in every task, highlighting the importance and effectiveness of MOHE for multi-omics feature learning. This indicates that MOHE can comprehensively utilize the complementary information across different omics modalities. This capability is underpinned by the fused hyperedge groups in the hypergraph learning process. Furthermore, after adding MOSA on MOHE_NN and NN_NN, the performance can be further improved on all three tasks, which indicates the effectiveness of MOSA. For example, on the BRCA dataset, the ACCs of MORE and NN_MOSA were 1.2% and 0.5% higher than those of MOHE_NN and NN_NN, respectively. It is noteworthy that the performance metrics of NN_MOSA and NN_NN were very close to each other on the GBM dataset. One possible reason is that the smaller number of disease categories and sample size may lead to the amplification of noise or errors in the absence of MOHE, thus potentially impacting the performance of MOSA. Nevertheless, the ablation study indicated that all the proposed modules in MORE are effective and can work synergistically.

**Table 3:** Ablation study of the key components on the ROSMAP and BRCA datasets.

Dataset		Evaluation metrics		
ROSMAP	Method	ACC	F1	AUC
	NN_NN	0.786 ± 0.022	0.790 ± 0.019	0.855 ± 0.019
	NN_MOSA	0.794 ± 0.020	0.799 ± 0.021	0.864 ± 0.022
	MOHE_NN	0.820 ± 0.020	0.825 ± 0.021	0.892 ± 0.021
	MORE	0.829 ± 0.018	0.836 ± 0.017	0.903 ± 0.010
**BRCA**	**Method**	**ACC**	**F1_weighted**	**F1_macro**
	NN_NN	0.797 ± 0.019	0.777 ± 0.021	0.726 ± 0.017
	NN_MOSA	0.802 ± 0.021	0.786 ± 0.024	0.731 ± 0.022
	MOHE_NN	0.823 ± 0.017	0.818 ± 0.020	0.757 ± 0.019
	MORE	0.835 ± 0.020	0.820 ± 0.023	0.768 ± 0.021

### Model performance under different omics settings

**Figure 3:**
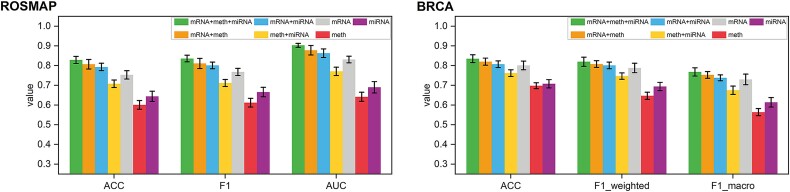
Classification performance with multi-omics data and single-omics data on the ROSMAP and BRCA datasets.

Within this subsection, we utilized three kinds of omics data: mRNA, meth, and miRNA. To verify the necessity of multiple omics integration for promoting model effectiveness, we particularly performed experiments under mRNA + meth + miRNA, mRNA + meth, mRNA + miRNA, meth + miRNA, mRNA, meth, and miRNA settings. As shown in Fig. 3 and Fig. S1, MORE integrating three kinds of omics data outperformed all other models in the aspect of ACC, F1, and AUC. Generally, MORE integrating two kinds of omics data outperformed that with only one kind of data. An exception was observed that the model integrating meth and miRNA performed worse than that using only mRNA, possibly due to the significant role of mRNA in the classification task. It is worth noting that the model integrating mRNA and meth on ROSMAP and BRCA dataset performed second best after integrating three kinds of omics data. However, on the GBM dataset, the model integrating mRNA and miRNA ranked the second best, suggesting that different classification tasks might require different combinations of omics data. Nevertheless, MORE that integrated all three kinds of omics data always attained the best performance in all tasks, further demonstrating the necessity and significance of multi-omics integration in various biological analyses.

### Model performance with different hyper-parameter

**Figure 4:**
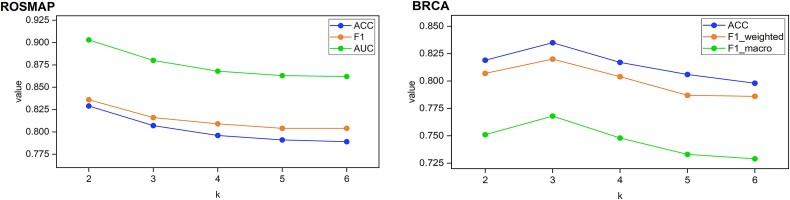
Classification performance of MORE with different hyper-parameters on the ROSMAP and BRCA datasets.

A crucial hyper-parameter in MORE is $k$, denoting the average number of nodes connected by each hyperedge. When constructing a hypergraph, if only a small amount of vertices are connected by each hyperedge, the hyperedge groups may become too sparse, thereby missing important connections among samples. Conversely, if each hyperedge connects too many vertices, the hyperedge groups may become too dense, potentially introducing noise into the correlation analysis among samples. Therefore, the choice of the proper value of $k$ is crucial for model performance. However, the optimal $k$ value depends on the topology of the dataset and varies across different datasets. To assess the effect of $k$ on MORE, we tested the proposed model on three datasets with different $k$ values. As can be seen from Fig. 4 and Fig. S2, different values of $k$ resulted in various performances. Particularly, on the ROSMAP dataset, MORE performed best when $k=2$, while on the BRCA and the GBM datasets, it achieved the best performance based on $k=3$. A possible explanation is that the AD classification task is a binary classification task with a relatively small amount of disease categories, thus requiring a smaller optimal $k$ value than multi-class classification tasks. In addition, the small sample size may also have an influence on the optimal $k$ value.

### Model performance based on different training strategies

Within this subsection, we utilized a two-step training strategy. During the pretraining phase, we trained each individual omics-specific MOHE module separately to initialize the model parameters. Subsequently, during the formal training process, we trained both MOHE and MOSA modules for final classification using multi-omics data. To prove the superiority of our strategy, we compared the performance based on the end-to-end training strategy with that of the two-step training strategy. From Table 4 and Table S2, we discovered that our training strategy performed better than end-to-end training. For example, the ACC of MORE on the ROSMAP dataset was 1.4% higher than that of the end-to-end training model. Moreover, on the BRCA dataset, all the performance metrics of MORE were higher than the end-to-end training ones, especially on F1_macro with a 2.1% improvement. These results adequately demonstrated the feasibility and effectiveness of our training strategy.

**Table 4:** Classification performance with different training strategies on the ROSMAP and BRCA datasets.

Dataset		Evaluation metrics		
ROSMAP	Strategy	ACC	F1	AUC
	End-to-end	0.815 ± 0.023	0.819 ± 0.023	0.885 ± 0.017
	Two-step (ours)	0.829 ± 0.018	0.836 ± 0.017	0.903 ± 0.010
**BRCA**	**Strategy**	**ACC**	**F1_weighted**	**F1_macro**
	End-to-end	0.819 ± 0.017	0.810 ± 0.020	0.747 ± 0.025
	Two-step (ours)	0.835 ± 0.020	0.820 ± 0.023	0.768 ± 0.021

## Identifying biomarkers using MORE

Biomarkers are defined as biochemical indicators for physiological, pathological, or therapeutic processes that can be objectively measured and evaluated [43, 44]. Accordingly, identifying biomarkers is critical for interpreting the trained deep neural networks and understanding the underlying molecular mechanisms [45, 46]. Within this subsection, we first introduce the employed theory for biomarker identification following with the discussion of the results in detail.

### Methods for identifying biomarkers

As the values of input features to MORE were scaled to $\left[0,1\right]$ during data preprocessing, we could eliminate particular features by changing its value to 0. In this way, the effect of that particular feature on the predictive performance could be measured by calculating the accuracy fluctuation after its removal. This method has been widely used in neural networks for feature importance grading [47, 48]. Through this method, we explored the importance of features from different kinds of omics data. When the classification performance decreases significantly after eliminating a feature, it is considered an important biomarker in this task. We utilized F1 and F1_macro to assess the performance of MORE for binary and multi-class classification tasks, respectively. We ran the experiment on the dataset five times and summed the classification performance decrease across these repetitions for each feature as the final indicator to ensure our results were reliable. Tables 5 and 6 provide the list of the identified top 10 biomarkers for each omics modality from ROSMAP and BRCA. GBM (used as a proof-of-concept) is not further analyzed for detailed biomarker identification. In order to prove the reliability of MORE, the inner product regularization [16] was also applied to the feature indicator matrix for selecting critical biomarkers. The outcomes, presented in Tables S3 and S4, demonstrate a high degree of consistency between the biomarkers identified by the two methods, confirming the robustness of MORE. An additional comprehensive explanation is provided in Text S3.

**Table 5:** Important biomarkers identified by MORE on the ROSMAP dataset.

Omics type	Top 10 important biomarkers
mRNA expression	ARRDC2, CDK18, KIF5A, CXCR4, NPNT, LNCBRM, CNN3-DT, QDPR, TCEA3, APLN
DNA methylation	MBOAT7, DNAJC16, TMC4, PCDH12, CCL3, ABCB5, FGD4, RAB34, AGA, TM4SF18
miRNA expression	hsa-miR-132, hsa-miR-146b-5p, hsa-miR-33a, hsa-miR-129-5p, hsa-miR-UL70-3p, hsa-miR-143, hsa-miR-133a, hsa-miR-129-3p, hsa-miR-374a, hsa-miR-640

**Table 6:** Important biomarkers identified by MORE on the BRCA dataset.

Omics type	Top 10 important biomarkers
mRNA expression	SOX11, GART, NRTN, PGBD5, PI3, AKR1E2, SLC6A14, KRT6B, CPA4, MASTL
DNA methylation	ADAMTSL5, NFIL3, ATP10B, LIMK1, PABPC4L, TFF3, DLGAP5, PAPPA2, CAMK2N1, SNORD21
miRNA expression	hsa-mir-187, hsa-mir-205, hsa-mir-130b, hsa-mir-451, hsa-mir-215, hsa-mir-503, hsa-mir-1269, hsa-mir-577, hsa-mir-526b, hsa-mir-204

For the most significant genes in mRNA and meth data, we conducted gene set functional enrichment analysis using the ToppGene Suite [49]. GO terms can be found through the ToppGene Suite. To make the results more convincing, we utilized the Benjamini–Hochberg procedure and reported the modified *P*-values. The biomarkers identified by MORE showed variation in their biological enrichment process and function across each dataset.

### Identified biomarkers associated with Alzheimer’s disease

For the biomarkers identified using mRNA expression data, significant enrichment was observed for GO terms relevant to CDK18 and APLN, such as cyclin-dependent protein kinase activity (GO: 0097472, *P* = 1.012E-2) and apelin receptor binding (GO: 0031704, *P* = 2.062E-2). According to earlier research, cyclin-dependent kinase 18 is bound by a cytosolic group of PLCβ, which facilitates tau phosphorylation and aggregation. Furthermore, by combining its catalytic activity and association with cyclin-dependent kinase 18, the PLCβ will lose and facilitate AD [50]. Additionally, Luo *et al*. [51] discovered that altering the amount of apelin can influence the course of neurodegenerative events like AD, indicating that apelin can become an ideal target to treat neurodegenerative diseases. Specifically, apelin’s effects include the suppression of apoptosis, reduction of oxidative stress, inhibition of Ca2+ signaling, induction of autophagy, and suppression of inflammatory response. Furthermore, positive regulation of neuroinflammatory response (GO: 0150078, *P* = 7.290E-2) and positive regulation of microglial cell migration (GO: 1904141, *P* = 1.861E-2) were found to be significantly enriched for the biomarkers identified by DNA methylation data. Shao *et al*. [52] reported that due to an elevated concentration of neuroinflammatory cytokines in AD, neuroinflammatory plays a role in the pathophysiology of the disease. Additionally, research has demonstrated that the central nervous system’s native innate immune cell population comprises microglia cells [53]. Microglial cell migration is thus closely linked to the progression of AD.

Moreover, in the AD patient classification task, some most significant biomarkers that were shown to be related to AD were also identified by MORE. Among the biomarkers identified by mRNA data, KIF5A has emerged as a possible candidate gene for regulating AD development. An essential component of the molecular machinery that facilitates anterograde axonal mitochondrial transport is kinesin-1, of which KIF5A is an isoform [54]. Studies have shown that brain mitochondrial defect is a significant feature of AD. Therefore, protecting the function of KIF5A is a potential treatment approach. Additionally, it is established that the chemokine CXC motif receptor4 (CXCR4) plays a role in the progression of AD. The etiology of AD involves complex factors, such as inflammation caused by microglia overactivation, and the expression of CXCR4 is elevated in astrocytes and microglia [55, 56]. For the biomarkers recognized by meth data, Hohman *et al*. [57] demonstrated an association between TMC4 and the development of AD. Yu *et al*. [58] reported a potential link between FGD4 and AD, verifying that FGD4 has a connection to actin cytoskeleton regulating mechanisms and may regulate synaptic loss in AD brain tissue. For the biomarkers identified by miRNA expression data, Nagaraj *et al*. [59] characterized different expressions of hsa-miR-33a in the plasma of AD and non-AD patients.

### Identified biomarkers associated with breast cancer

For the biomarkers identified by mRNA data, we discovered some GO terms relevant to breast cancer were significantly enriched, including solute: sodium symporter activity (GO: 0015370, *P* = 8.117E-3) and positive regulation of osteoblast differentiation (GO: 0045669, *P* = 2.267E-2). It has been shown that sodium symporter protein is widely expressed in breast tumors, indicating the potential for breast cancer radiation treatment [60]. Additionally, Wu *et al*. [61] found that osteoblasts can deposit collagens to suppress Natural Killer (NK) cells through the inhibitory LAIR1 signaling and stimulate breast tumor colonization. Moreover, Adenosine Triphosate (ATP) dependent activity (GO: 0140657, *P* = 5.500E-2) was greatly enriched among the biomarkers identified by meth data. Studies have suggested that ATP-dependent activity is involved in breast cancer by showing the overexpression of ATPase phospholipid transporting 10B in breast cancer cells [62].

For invasive breast carcinoma subtype classification, there were also certain most significant biomarkers identified by MORE, which have proven to be associated with breast cancer. For the biomarkers identified by mRNA data, SOX11 has been shown to be associated with invasive cancer development. SOX11, usually inactive in mammary cells after birth, is expressed in estrogen receptor-negative Ductal Carcinoma In Situ (DCIS) lesions, particularly in basal-like clusters with increased aldehyde dehydrogenase activity and mammosphere formation capacity, and studies confirmed that SOX11 promotes the progression of DCIS to invasive cancer [63, 64]. In addition, Dunlap *et al*. [65] found that PI3 can promote the development of invasive breast carcinoma. For the biomarkers identified by meth data, TFF3 is strongly linked to invasive breast carcinoma. Studies showed that TFF3 is highly expressed in fibrocystic changes and papillomatous areas, with 89% expression in carcinomas *in situ* and 83% in invasive carcinomas [66, 67]. After analyzing tissue samples from individuals with invasive ductal carcinoma of the breast, Dietrich *et al*. [68] identified LIMK1 as a biomarker for invasive breast carcinoma. For the biomarkers identified by miRNA data, Gupta *et al*. [69] discovered that hsa-mir-503 is expressed in many types of tumors, like breast cancer and hepatocellular carcinoma. Their research demonstrated that hsa-mir-503 exerts its tumor-suppressing effect through its action on target genes. Xiao *et al*. [70] investigated the relationship between hsa-mir-205 and breast cancer and found that the gene has a tumor suppressor effect.

## Conclusion

Recent advances in multi-omics sequencing techniques have enabled multiview characterization of various complex biological processes and diseases. Herein, we have proposed an innovative multi-omics integration method, termed MORE, for a more efficient and accurate multi-omics analysis. Specifically, MORE is developed based on two major modules MOHE and MOSA, which are designed to extract the omics-specific features and integrate the multiple omics information, respectively. The effectiveness of MORE was benchmarked on three classification tasks. The results showed that it significantly outperformed several existing integrated multi-omics approaches. The ablation study further demonstrated the significance and contribution of each proposed module during the analysis process. Furthermore, we found that the model combining three kinds of omics data had the best classification performance, proving the value of integrating diverse omics data. Additionally, MORE was effective in identifying potential biomarkers associated with various diseases, including AD, invasive breast carcinoma, and glioblastoma, and also had excellent capability in model interpretation. Despite the advantages, the proposed MORE method has certain limitations in terms of its capability of modeling other data modalities, e.g. medical image data and clinical reports. In further research, we aim to design a more robust and comprehensive framework to facilitate disease-oriented multi-omics data processing.

**Key Points**

- We propose an innovative multi-omics integration method termed “MORE” to enable the integration of different omics data modalities for efficient analysis.
- MORE utilizes the MOHE module to effectively learn omics-specific features and the MOSA module to integrate valuable information across different data modalities, respectively.
- The core component of the MOHE module is a hypergraph, which can encode higher-order data correlations with its degree-free hyperedges. The MOHE module can simultaneously reveal correlations within and across omics modalities, and the MOSA module considers the varied contributions of diverse omics modalities to the last forecast.
- We verify the excellent performance of MORE in contrast with several current superior methods through extensive benchmarking experiments.
- We show the predictive power of MORE in effectively identifying disease-related biomarkers, highlighting its excellent biomedical data mining and interpretation capabilities.

## Supplementary Material

Supplementary_material_final_version_bbae658
