# Supplementary material for: MORE: a multi-omics data-driven hypergraph integration network for biomedical data classification and biomarker identification
Source: Brief Bioinform. 2024 Dec 18;26(1):bbae658. doi: 10.1093/bib/bbae658 (PMC11653202; doi:10.1093/bib/bbae658)
Supplement: Supplementary_material_final_version_bbae658 [file supplementary_material_final_version_bbae658.docx]

**MORE: a multi-omics data-driven hypergraph integration network for biomedical data classification and biomarker identification**

Yuhan Wang^1,#^, Zhikang Wang^2,#^, Xuan Yu^3^, Xiaoyu Wang^2^, Jiangning Song^2,4,*^, Dong-Jun Yu^1,*^, and Fang Ge^5,*^

^1^School of Computer Science and Engineering, Nanjing University of Science and Technology, 200 Xiaolingwei, Nanjing, 210094, China;

^2^Monash Biomedicine Discovery Institute and Department of Biochemistry and Molecular Biology, Monash University, Melbourne, VIC 3800, Australia;

^3^Department of Computer Science, City University of Hong Kong, Kowloon, Hong Kong, 999077, China;

^4^Monash Data Futures Institute, Monash University, Melbourne, VIC 3800, Australia;

^5^State Key Laboratory of Organic Electronics and Information Displays & Institute of Advanced Materials (IAM), Nanjing University of Posts & Telecommunications, 9 Wenyuan, Nanjing, 210023, China.

^#^ The first two authors contributed equally to this work and should be considered as co-first authors.

^*^ To whom correspondence should be addressed.

Corresponding authors: Fang Ge, [gfang0616@njupt.edu.cn](mailto:gfang0616@njupt.edu.cn); Dong-Jun Yu, [njyudj@njust.edu.cn](mailto:njyudj@njust.edu.cn); Jiangning Song, [jiangning.song@monash.edu](mailto:jiangning.song@monash.edu).

**Supplementary Texts**

**Text S1: Performance evaluation**

**Accuracy (ACC):** ACC represents the proportion of correctly predicted samples to all samples, where there may be positive or negative samples correctly predicted. The ACC is calculated as follows:

$$ACC=\frac{TP+TN}{TP+TN+FP+FN}$$

where TP is true positive, TN is true nNegative, FP is false positive, and FN is false negative.

**F1 score (F1):** The F1 score combines the two metrics Precision and Recall. When the value of F1 is 1, it means that the model is the most effective, and when the value is 0, it means that the model is the least effective. Precision indicates the proportion of true positive samples in the predicted positive samples. Recall indicates the proportion of predicted true positive samples in all true positive samples. The two indicators can be calculated as follows:

$$Precision=\frac{TP}{TP+FP}$$

$$Recall=\frac{TP}{TP+FN}$$

The F1 score is defined as:

$$F1=\frac{2Precision\times Recall}{Precision+Recall}$$

**Area under the receive operating characteristic curve (AUC):** AUC represents the area under the ROC curve, which can be interpreted as the overall ability of the model to discriminate true samples. When the value of AUC is 1, it means that the model works best, and the value of 0.5 means that the model does not work. The ROC curve takes FPR as the horizontal axis and TPR as the vertical axis, where FPR represents the false positive sample rate, and TPR represents the true positive sample rate. The FPR and TPR can be calculated as:

$$FPR=\frac{FP}{FP+TN}$$

$$TPR=\frac{TP}{TP+FN}$$

**Average F1 score weighted by support (F1_weighted):** Calculating F1_weighted requires distinguishing categories, calculating the F1 score for each category, and taking a weighted average of the F1 scores of all the categories, with the weights being the proportion of samples corresponding to each category to the total number of samples.

**Macro-averaged F1 score (F1_macro):** Calculating F1_macro requires distinguishing categories, calculating Precision, Recall, and F1 score for each category, and averaging the F1 score for all categories.

**Text S2: MORE implementation**

MORE was implemented on PyCharm using PyTorch 1.9.1 and Python 3.9, with the Windows 10 system environment. MORE was divided into two steps: pre-training and formal training. For pre-training, only the MOHE was trained. We set the pre-training process to 500 epochs and the learning rate of the MOHE was set to 1e-3. Formal training was performed by training the MOHE and MOSA. The learning rate of the MOHE and MOSA were set to 5e-4 and 1e-3, respectively. The formal training process was set to 1500 epochs. MOHE and MOSA used the Adam optimizer throughout.

**Text S3: Different methods for identifying biomarkers**

Two distinct methods, MORE and the approach described in Ref. [1], were employed to identify key biomarkers. MORE utilizes feature importance scores, while Ref. [1] applies inner product regularization to the feature indicator matrix. MORE assesses the contribution of individual features to predictive performance by systematically setting their values to zero and observing the resulting changes in accuracy. Features whose exclusion significantly reduces classification performance are identified as critical biomarkers. To enhance the robustness of the analysis, MORE was complemented by the inner product regularization approach, which incorporates feature interactions into the selection process by constraining the feature indicator matrix.

The results, summarized in **Tables 5–6** and **Tables S3–S4**, reveal strong concordance between the two methods, with only minor discrepancies. For instance, on the ROSMAP dataset, both MORE and Ref. [1] consistently identified key biomarkers such as ARRDC2, CDK18, CCL3, TMC4, hsa-miR-33a, and hsa-miR-132. Discrepancies were limited to two low-ranking mRNA biomarkers. Similarly, on the BRCA dataset, only one biomarker differed in the DNA methylation analysis. These findings confirm the reliability and robustness of MORE while demonstrating its compatibility with the inner product regularization approach.

**Supplementary Figures**

**
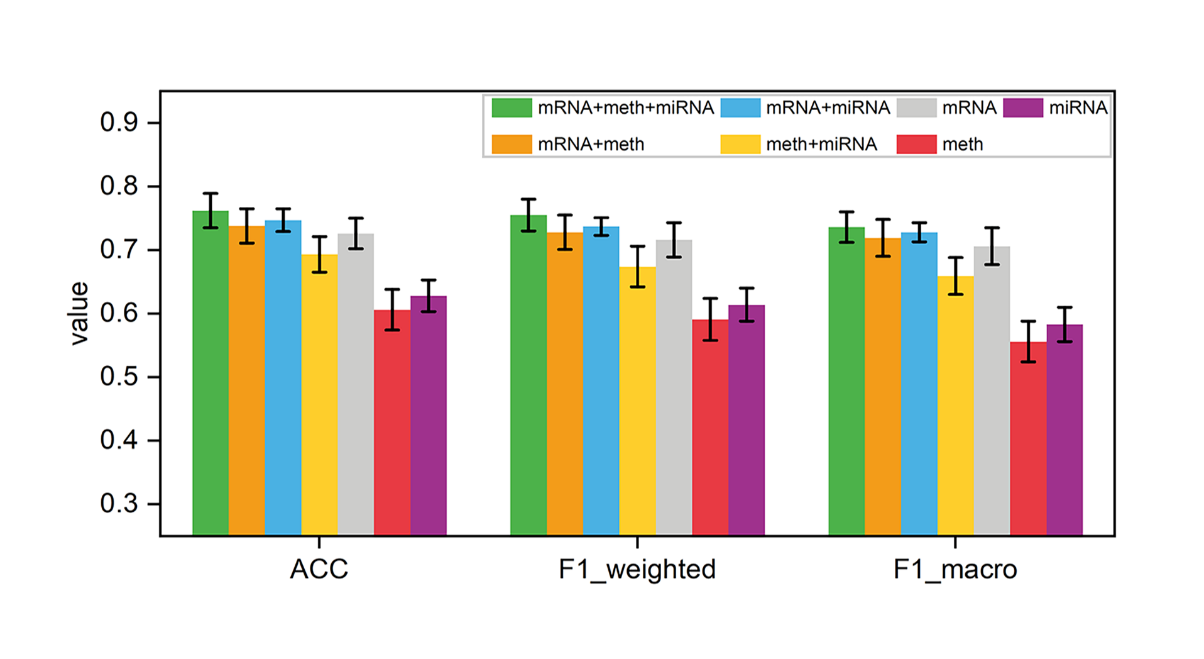
**

**Figure S1.** Classification performance with multi-omics data and single-omics data on the GBM dataset.


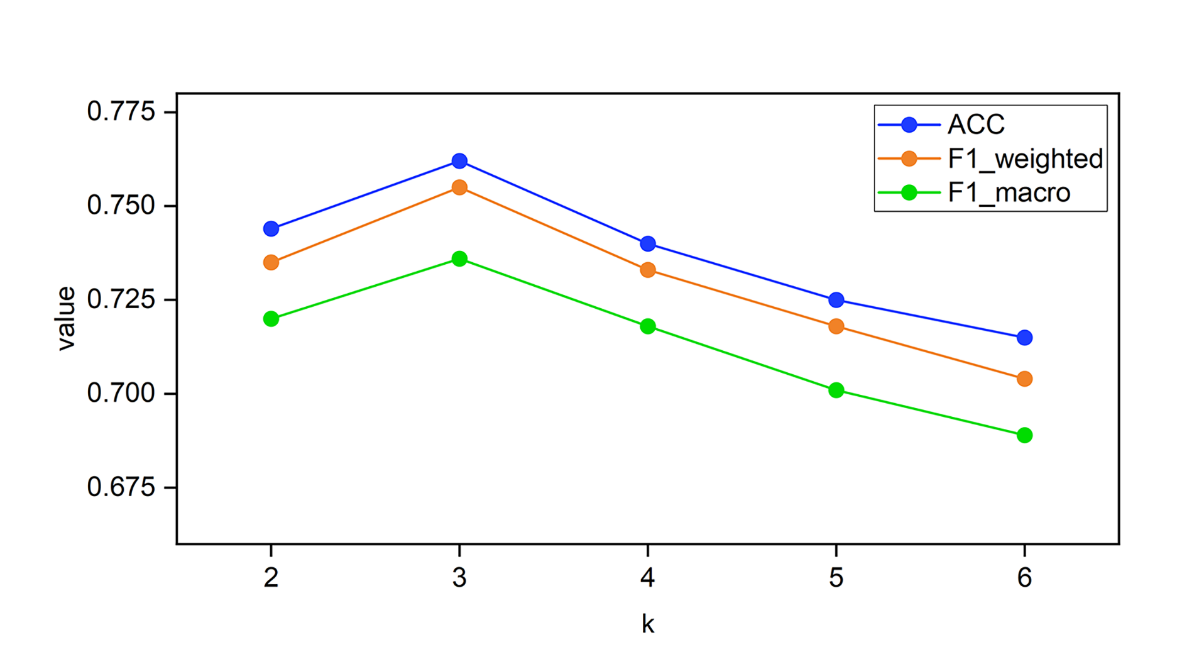


**Figure S2.** Classification performance of MORE with different hyper-parameter on the GBM dataset.

**Supplementary Tables**

**Table S1.** Ablation study of the key components on the GBM dataset

| Method | ACC | F1_weighted | F1_macro |
| --- | --- | --- | --- |
| NN_NN | 0.724±0.026 | 0.704±0.021 | 0.685±0.023 |
| NN_MOSA | 0.726±0.020 | 0.706±0.017 | 0.686±0.025 |
| MOHE_NN | 0.746±0.025 | 0.738±0.025 | 0.728±0.022 |
| MORE | **0.762±0.027** | **0.755±0.025** | **0.736±0.024** |

**Table S2.** Classification performance with different training strategies on the GBM

dataset

| Dataset |  | Evaluation metrics | | |
| --- | --- | --- | --- | --- |
| GBM | Strategy | ACC | F1_weighted | F1_macro |
|  | end-to-end | 0.744±0.023 | 0.735±0.027 | 0.725±0.023 |
|  | two-step (ours) | **0.762±0.027** | **0.755±0.025** | **0.736±0.024** |

**Table S3.** Important biomarkers identified by inner product regularization of the feature indicator matrix on the ROSMAP dataset

| Omics type | Top 10 important biomarkers |
| --- | --- |
| mRNA expression | ARRDC2, CDK18, KIF5A, PLXNB1, CXCR4, NPNT, LNCBRM, APLN, AQP6, TCEA3 |
| DNA methylation | CCL3, TMC4, MBOAT7, FGD4, DNAJC16, TM4SF18, GBGT1, AGA, ABCB5, RAB34 |
| miRNA expression | hsa-miR-132, hsa-miR-133a, hsa-miR-146b-5p, hsa-miR-640, hsa-miR-143, hsa-miR-33a, hsa-miR-129-3p, hsa-miR-374a, hsa-miR-206, hsa-miR-129-5p |

**Table S4.** Important biomarkers identified by inner product regularization of the feature indicator matrix on the BRCA dataset

| Omics type | Top 10 important biomarkers |
| --- | --- |
| mRNA expression | SOX11, PI3, GART, SLC6A14, PGBD5, ECE2, KRT6B, TCAM1P, NRTN, BCL11A |
| DNA methylation | LIMK1, ADAMTSL5, NFIL3, DARC, PABPC4L, TFF3, DLGAP5, PAPPA2, CAMK2N1, ATP10B |
| miRNA expression | hsa-mir-187, hsa-mir-204, hsa-mir-15b, hsa-mir-451, hsa-mir-205, hsa-mir-215, hsa-mir-503, hsa-mir-1269, hsa-mir-656, hsa-mir-526b |

**Reference:**

[1] D. Ouyang, Y. Liang, L. Li *et al.*, Integration of multi-omics data using adaptive graph learning and attention mechanism for patient classification and biomarker identification. *Comput Biol Med* **164**, 107303 (2023).
